# Influence of age, gender, and prodromal symptoms on sudden death in a tertiary care hospital, eastern Saudi Arabia

**DOI:** 10.4103/1319-1683.71989

**Published:** 2010

**Authors:** Houssien Kamal Nofal, Mohammed Fakhry Abdulmohsen

**Affiliations:** *Department of Pathology/Forensic Medicine, College of Medicine, University of Dammam, Dammam, Kingdom of Saudi Arabia*; 1 *Department of Internal Medicine, College of Medicine, University of Dammam, Dammam, Kingdom of Saudi Arabia*

**Keywords:** Sudden death, sudden cardiac death, unexpected sudden death, expected death, coronary artery disease, atherosclerotic coronary heart disease, acute myocardial infarction, sudden infant death syndrome

## Abstract

**Background:**

Sudden death (SD) remains an important worldwide public health problem. The incidence of SD and causes vary in different societies, and these differences are influenced also by demographic and clinical factors such as age, gender and prodromal symptoms and signs. This six-year study describes the influence of these factors on SD.

**Materials and Methods:**

This is a retrospective study of SD in all age groups undertaken in King Fahd Hospital of the University (KFHU), Eastern Saudi Arabia. All cases of death (1273 total, 1050 expected death and 223 cases of sudden unexpected death) that occurred between January 1, 2000 and December 31, 2005 were investigated and subsequently analyzed on demographic and clinical parameters of the deceased patients. The statistical analysis was performed as appropriate to illustrate any possible association between different demographic variables and SD.

**Results:**

There were 223 cases of SD (17.5%) out of 1273 total deaths in KFHU in the 6-year study period. There was a definite influence of age on the incidence of sudden death (SD) as it increased clearly at the two ends of the age spectrum, 32.2% of the cases were infants (from birth to 12 months), and 31.4% were elderly (> 60 year-old). However, among infantile age group, the highest frequency of SD (22.2% of the cases) was among the neonates. There was also a significant trend of gender influence on the incidence of SD which was higher in men than women (56% vs. 42%). The influence of prodromal symptoms and signs on SD was variable. Dyspnea and cough as major symptoms of cardiovascular and respiratory disease were the most frequent presenting symptoms in 32.3% of the cases, followed by fever as a sign of infections in 11.7%, premature infants in 10.8%, circulatory collapse in 9.4%, and angina in 7.6% of the cases.

**Conclusion:**

The current study indicated a definite influence of age, gender and prodromal symptoms on the incidence of SD. The highest incidence occurred in the two extremes of age scale as compared to other age groups. Incidence was also higher in men than women. Meanwhile, the major prodromal symptoms and signs were dyspnea and cough, fever, premature birth, circulatory collapse, and angina pectoris..

## INTRODUCTION

The World Health Organization (WHO) defines sudden death (SD) according to the International Classification of Diseases (ICD-10) as non-violent death that occurs in less than 24 hours from the onset of the symptoms.[1] The problem of sudden death is still controversial with respect to its causes and relation to many risk factors such as age, gender, prodromal symptoms, environment, nutrition and stress. It can be instantaneous or not, and it can occur at home or in the street, in bed during sleep or even on a vehicle.[1–7] SD occurs in all age groups such as infants (0-1 year), childhood and adolescence (1-17years), young adults (18-39), middle age (40-60), and elderly age group (>60 years).[7–10] It is also well known that causes of SD depend on age, gender, ethnicity and genetics.[9–12] Therefore, a history of prior syncope, especially if there is recurrence, a family history of premature sudden cardiac death (SCD), a critical coronary artery lesion, an abnormal electrocardiogram with prolonged QTc > 450msec or with the features of Brugada Syndrome, poor left ventricular contractile function (e.g., EF<35%), or echocardiographic features hypertrophic cardiomyopathy (HCM), and arrhythmogenic right ventricular dysplasia/cardiomyopathy (ARVD/C) should alert the physician about the risk of SCD.[11] Prevention of SD is a very important medical task, and ultimately the up-to-date medical knowledge about causes and risk factors of SD is extremely important to improve the attitude and practice of health care providers in order to reduce the magnitude of this serious problem. Studies on the problem of SCD have originated mainly from North American and Europe, since it is considered a major public health problem in western societies that accounts for 20% of all mortality.[12] SCD rates increased in the USA by 12.4% between 1989 and 1998 (from 56.3% to 63.9%). The age adjusted SCD rates declined 11.7% in men and 5.8% in women.[13] Compared to men, women are less likely to suffer from SCD and surprisingly women who have SCD are less likely to have had prior history of heart disease (37% vs. 56%). In individual subjects’ ≥40 year’s old, atherosclerotic coronary heart disease (ASHD) is the most common cause of SCD.[14] Between one and 40 years of age, the causes of SCD are commonly hypertrophic cardiomyopathy (HCM), myocarditis, congenital heart disease, and less common causes include aortic dissection, valvular heart disease, and arrhythmogenic right ventricular dysplasia (ARVD).[10–17]

In a Spanish study on a series of 107 SD cases with ages ranging between one and 35 years from 1991 to 1998, Morentin and his colleagues found that the mortality rate was 2.4/100.000.[18] Men were at a three-fold risk for SD in contrast to women. About 43% of SD cases in this series were due to SCD, and ASHD was the most frequently found in those over the age of 30 years. Myocardial disease and conduction system abnormalities were common between 15 and 30 years of age. Of the noncardiac causes such as infections which were frequently noted in children, epilepsy, asthma and intracranial hemorrhage represented 39% of the cases, occurring mainly in those between 15 and 30 years of age. Notably, 18% of the cases, especially children, had a negative postmortem.[19] Under the age of one year, SCD can be part of sudden infant death syndrome (SIDS)[8,19–21] which is usually associated with a negative postmortem, the cause of which is most likely to be a primary arrhythmogenic event.[12] However, the incidence of SIDS has significantly declined, from 1 to 6 cases per 1000 live births before 1991 to 0.5 to 1.5 cases per 1000 live births.[20,21] In a retrospective study including 162 cases of unexpected sudden death in a relatively young age groups between 9 and 39 years, Drory and his colleagues[22] found that the main underlying causes of SD were cardiovascular disease in 73 % of the cases, noncardiac disease in 15%, and in 12% the cause was unidentifiable. In patients younger than 20 years, 22% suffered from myocarditis, 22% from hypertrophic cardiomyopathy (HCM), and 13% suffered from conduction system abnormalities. In those between 20 and 29 years, 24% had CHD, 22% myocarditis, and 13% had HCM. Whereas, in those aged 30 or above, 58% suffered from CHD and 11% from myocarditis. Noncardiac causes included intracranial hemorrhage in 5% of the cases and infections in 4%. Prodromal symptoms were reported by 54% of subjects, chest pain in 25% of patients aged 20 years or above, dizziness in 16% of patients below 20 years. SD occurred in 49% of the cases while engaged in routine daily life activity, and in 23%, they occurred during sleep.[22]

This retrospective analysis of SD was done during the six-year study period to elucidate the possible influence of age, gender and prodromal symptoms on the incidence of SD in a large University Hospital (500 bed hospital), Eastern Region, Saudi Arabia.

## MATERIALS AND METHODS

This is a retrospective clinical study of sudden death (SD) in all age groups, undertaken in KFHU, Eastern Saudi Arabia. All cases of deaths (1273 cases) that occurred between January 1, 2000 and December 31, 2005 were investigated. There were 223 cases of SD in less than 24 hours from the onset of symptoms of their final clinical presentations. From all cases, identity and demographic data, history of preexisting diseases, major symptoms (chief complaints) on presentation, clinical signs, performed medical investigations, diagnosis of death, and the time lapse between the onset of initial symptoms at presentation and the onset of death were obtained from the medical files.

Permission was sought from the research committees of the College of Medicine, University of Dammam, Dammam, and KFHU, Al-Khobar, Kingdom of Saudi Arabia. The age and gender differences and the prodromal symptoms and signs were statistically analyzed to find if they influenced in any way the incidence of SD. Data were entered in computer using SPSS for windows version 13.0 (SPSS Inc., Chicago, IL). Results were cross-tabulated to find out the relationships between the variables. Statistical analysis was performed using χ^2^-square for test of association and Fisher’s exact test as appropriate. A p-value of less than 0.05 was considered significant in all statistical analysis.

## RESULTS

**Figure 1:**
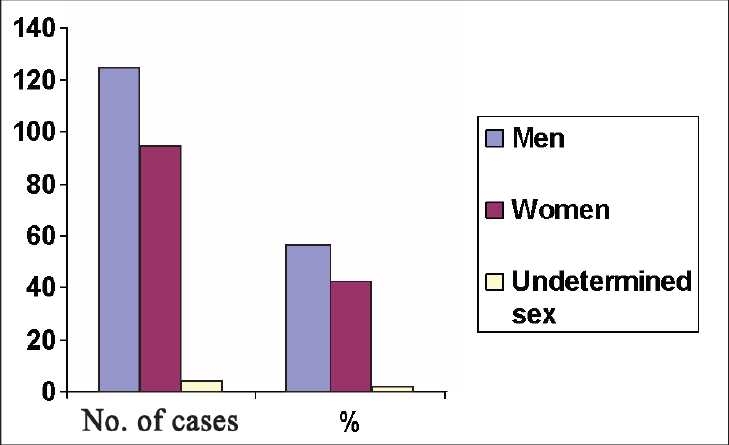
The effect of gender on the incidence of SD during the 6-year study period.

Our study revealed a definite age influence on the incidence of SD. Table 1 illustrates clearly that the incidence of SD increases a great deal in the two age extremes. In newborns (from birth to 7 days), there were 51 out of 223 cases (22.9%), 6 cases (2.7%) in the neonatal age group (1 to 4 weeks of age), 15 cases (6.7%) in infants between 1 and 12 months, and collectively in infant age group (from birth to 12 months), there were 72 cases of SD (32.3%). Moreover, there were 70 cases of SD (31.4%) in the elderly (age >60 years). The lowest incidence of SD, 6 cases (2.7%) occurred in children aged 1 to 11years, and in adolescents aged 12 to 18 years, 6 cases (2.7%). The incidence of SD increased slightly, 22 cases (9.9%), in young adults aged 19 to 39 years, but there was a significant increase, 47 cases (21.1%), in the middle age group (from 40 to 60 years), *P* < 0.0001. Figure 1 shows a strong trend of higher incidence of SD in men than women [125 cases (56%) vs. 94 cases (42%), *P*=0.237]. The undetermined sex who suffered SD during the study period was only 4 cases (1.8%). The frequency of prodromal symptoms and signs affecting cases of SD is presented in Table 2. Dyspnea as a cardinal cardiovascular and respiratory symptom was the most frequent prodromal symptom affecting 56 cases of SD (25.1%). Twenty-six cases (11.7%) had fever, 24 cases (9.4%) were premature infants, 21 cases (9.4%) presented with circulatory collapse, 17 cases (7.6%) with angina, 16 cases (7.2%) with cough, 12 (5.3%) cases were comatozed, 12 cases (5.3%) with anorexia, 10 cases (4.5%) with generalized weakness, 6 cases (2.7%) with seizures, 5 cases (2.2%) with diarrhea, 2 cases (0.9%) with abdominal distension, and 2 cases (0.9%) with dementia. The cardiovascular and respiratory symptoms including dyspnea, circulatory collapse, angina and cough affected almost half of the victims of SD, 110 cases (49.3%) in the current study.

**Table 1:** The influence of age on SD in KFHU during the 6-year study period.

Years	2000	2001	2002	2003	2004	2005	Total	%
Sudden deaths	47	35	37	43	26	35	223	100
0-7 D	7	8	11	12		13	51	22.9
1-4 W	2	2	2				6	2.7
1-12 M	4	3	1	1	4	2	15	6.7
0-12M	13	13	14	13	4	15	72	32.2*
1-11 Y	2	1		1	1	1	6	2.7
12-18 Y	2	2	1	1			6	2.7
19-39Y	6	4	5	1	5	1	22	9.9
41-60 Y	12	7	3	10	9	6	47	21.1
> 60 Y	12	8	14	17	7	12	70	31.4*

* *P* < 0.0001

**Table 2:** The most frequent prodromal symptoms and signs of cases of sudden death

Initial symptoms	No. of cases of SD	%
Dyspnea*	56	25.1
Fever	26	11.7
Premature infants	24	10.8
Circulatory collapse*	21	9.4
Angina*	17	7.6
Cough*	16	7.2
Coma	12	5.3
Anorexia	12	5.3
Generalized weakness	10	4.5
Seizures	6	2.7
Diarrhea	5	2.2
Abdominal distension	2	0.9
Seizures	2	0.9

* Cardiovascular and respiratory symptoms

## DISCUSSION

The current retrospective hospital-based study presents enough evidence of the influence of age and gender on the incidence of SD in this part of the world, as it was much higher at the two ends of the age spectrum than other age groups, and was also higher in men than in women. These findings were consistent with the international experience, especially the findings from western countries.[7–13,18–21,23,24] However, the incidence of SIDS as an important component of SD in infant age group has significantly declined over the last two decades, probably due to the improved medical care of this age group and the increased practice of family counseling.[9,19–21] The incidence of major cardiovascular and respiratory symptoms and signs was quite high affecting 49.3% of our patients (dyspnea in 25.1%, cough in 7.2%, circulatory collapse in 9.4%, and angina in7.6%). These findings reveal that presumably, there is a strong link between the prodromal symptoms and clinical signs of critically ill patients at the final presentation and their outcome, especially if their symptoms and signs are related to cardiac and/or respiratory medical emergency. Drory *et al*., reported that similar incidence of 54% of prodromal symptoms affected his SD cases. The two main prodromal symptoms were dizziness in the age group younger than 20 years, and chest pain in subjects aged more than or equal to 20 years.[22] In another study by Alonzo AA *et al*., of 160 hospitalized patients with acute myocardial infarction, and 138 individuals who had died prior to hospitalization from acute heart disease, it was found that 70% of the hospital subsample and 64% of the out of hospital subsample reported prodromal symptoms.[25] However, Engelstein ED and Zipes DP, stated that prodromal symptoms were often nonspecific, and could only be suggestive. They believed that there was age, race and sex influence on SCD, but the proportion of sudden coronary deaths decreased with age. The annual incidence of SCD among men was 3-4 times higher than in women, and the peak incidence of SCD occurring between birth and 6 months of age was essentially due to SIDS.[23] We strongly believe that prodromal symptoms and signs can be very helpful for the prevention of SD. For example, history of class III or class IV dyspnea, dizziness or syncopal attacks, very fast palpitation, or chest pain could direct the attention of the treating physician to request specific investigations such as electrocardiogram, echocardiogram, 24 Hours Holter Monitoring, coronary angiogram and if needed electrophysiological study to arrive at an accurate diagnosis and provide the appropriate treatment.[11,22,25] Nonetheless, a prompt appreciation of prodromal symptoms during that phase of a life threatening medical problem such as acute myocardial infarction by lay people and paramedical personnel is necessary if intervention by the treating physician is to be effective. It is also noteworthy to emphasize that a prompt utilization of time, a narrow, but very precious window of opportunity may save a lot of lives of patients presenting with life threatening cardiovascular, respiratory or neurological conditions.

## CONCLUSION

The current study illustrates a clear influence of age and gender on the incidence of SD. The incidence of SD increased significantly at the two extremes of age in infants and the elderly (< 12 months and >60years), and there was a strong trend of higher incidence in men than in women. Prodromal symptoms and signs reported by the majority of our SD individuals, their witnesses and the emergency physicians, had a probable strong link with the incidence of SD. Therefore, to improve on the response, attitude and practice of the prevention of SD, the awareness of the lay and medical communities with regard to the prodromal symptoms and signs of the phenomenon should be improved.

## References

[1] (2005). World Health Organization. International Classification of Diseases (ICD-10). WHO.

[2] Spiliopoulou C, Papadodima S, Kotakidis N, Koutselinis A (2005). Clinical diagnoses and autopsy findings: a retrospective analysis of 252 cases in Greece. Arch Pathol Lab Med.

[3] Morentin B, Paz Suarez-Mier M, Audicana C, Aguilera B, Manuel Garamendi P, Elexpe X (2001). Incidence and causes of sudden death in persons less than 36 years of age. Med Clin (Barc).

[4] Rossi S, Reale D, Grandi E (1991). Correlation of clinical diagnosis with autopsy findings. IARC Sci Publ.

[5] Kojima M, Kawamura T, Lin Y, Aoki R, Wakai K, Tamakoshi A, Ohno Y (1999). Sudden death of clinically unknown origin. An overview of postmortem examinations in Japan. Nippon Koshu Eisei Zasshi.

[6] Nashelsky MB, Lawrence CH (2003). Accuracy of cause of death determination without forensic autopsy examination. Am J Forensic Med Pathol.

[7] Blackwell CC, Busuttil A, Weir DM, Saadi AT, Essery SD (1994). Sudden unexpected nocturnal deaths among Thai immigrant workers in Singapore. The possible role of toxigenic bacteria. Int J Legal Med.

[8] Bubnaitiene; V, Kalediene R, Kevalas R (2005). Case-control study of sudden infant death syndrome in Lithuania, 1997-2000. BMC.

[9] Eckart RE, Scoville SL, Campbell CL, Shry EA, Stajduhar KC, Potter RN (2004). Sudden death in young adults: a 25-year review of autopsies in military recruits. Ann Intern Med.

[10] Di Maio VJM, Di Maio DJ (2001). Forensic Pathology.

[11] Sung RJ, Kuo Chi-Tai, Wu Shan-Nan, Lai WT, Luqman N, Chan NY (2008). Sudden Cardiac death syndrome: Age, Gender, Ethnicity, and Genetics. Acta Cardiol Sin.

[12] de Vreede-Swagemakers JJ, Gorgels AP, Dubois-Arbouw WI, van Ree JW, Daemen MJ, Houben LG (1997). Out-of-hospital cardiac arrest in the 1990’s: a population based study in the Maastricht area on incidence, characteristics and survival. J Am Coll Cardiol.

[13] Zheng ZJ, Croft JR, Giles WH, Menash GA (2005). Sudden Cardiac Death in United States, 1989 to 1998. Am J Prev Med.

[14] Kannel WB, Wilson PW, D’Agostino RB, Cobb J (1998). Sudden coronary death in women. Am Heart J.

[15] Neuspiel DR, Kuller LH (1985). Sudden and unexpected natural death in childhood and adolescence. JAMA.

[16] Anderson RE, Hill RB, Broudy DW, Key CR, Pathak D (1994). A population-based autopsy study of sudden unexpected deaths from natural causes among persons 5 to 39 years old during 12 years period. Hum Pathol.

[17] Shen WK, Edwards WD, Hammill SC, Bailey KR, Ballard DJ, Gersh BJ (1995). Sudden unexpected nontraumatic death in 54 young adults: a 30year population-based study. Am J Cardiol.

[18] Morentin B, Suarez-Mier MP, Aguilera B (2003). Sudden unexplained death among persons 1-35 years old. Forensic Sci Int.

[19] Burnett LB, Adler J (2001). Sudden Infant Death Syndrome. Medicine J.

[20] Fleming P, Blair P, Bacon C, Berry J (2000). Sudden unexpected deaths in infancy. The CESDI SUDI Study.

[21] Kaarene Fitzgerald AC SIDS Global Strategy Task Force: international SIDS statistics.

[22] Drory Y, Turetz Y, Hiss Y, Lev B, Fisman EZ, Pines A (1991). Sudden Unexpected Death in Persons less than 40 years. Am J Cardiol.

[23] Engelstein ED, Zipes DP, Alexander RW, Schlant RC, Fuster V (1998). Sudden Cardiac Death. The Heart, Arteries and Veins.

[24] Myeberg, Robert J (2005). Cardiac arrest and sudden cardiac death. In: Heart Disease. A Textbook of Cardiovascular Medicine.

[25] Alonzo AA, Simon AB, Feinleib M (1975). Prodromata of myocardial infarction and sudden death. Circulation.

